# Case Report: A possible novel adult-onset, progressive MAO-A hypofunction

**DOI:** 10.3389/fnins.2026.1743519

**Published:** 2026-02-25

**Authors:** Stefan Berteau, Erik Armitano

**Affiliations:** 1 Independent Researcher, Edinburgh, United Kingdom; 2 Complex Autonomic Center, Mountlake Terrace, WA, United States

**Keywords:** carvedilol, hypofunction, MAO-A, monoamine oxidase A, norepinephrine, plasma catechols, rasagiline

## Abstract

We describe the clinical presentation, pathophysiology, and successful treatment of a previously undocumented adult onset, progressive form of monoamine oxidase A hypofunction. The patient experienced progressive symptoms consistent with excess intracellular noradrenaline in the sympathetic nervous system and with a reduced ability to metabolize tyramine - both associated with low monoamine oxidase A activity. The nature of the pathophysiology was then tested first by fractionated plasma catecholamine assays, performed at baseline and again using entacapone challenge to suppress catechol-O-methyltransferase function. Plasma noradrenaline levels are unaffected by entacapone in healthy adults, due to monoamine oxidase A activity. This was followed by a direct measurement of plasma catechols (specifically metabolites of norepinephrine and dopamine), to compare their respective levels and ratios to known cases of X-linked monoamine oxidase A microdeletion. Under the entacapone challenge, the patient’s plasma noradrenaline increased by 89%, consistent with monoamine oxidase A hypofunction. When repeated with daily rasagiline administration, the increase fell to 14%. Further challenges showed a variable but consistent increase under entacapone. Direct measurement of catechols measurement showed that levels of dihydroxyphenylglycol, a metabolite of norepinephrine via monoamine oxidase A, and 3,4-dihydroxyphenylacetic acid, a metabolite of dopamine via monoamine oxidase A, were significantly below reference range, consistent with reduced monoamine oxidase A activity. Treatment consistent with the hypothesis consisted of rasagiline or selegiline combined with carvedilol. This treatment relieved all symptoms.

## Introduction

We describe a previously undocumented adult-onset, progressive form of monoamine oxidase A (MAO-A) hypofunction in a patient who is one of the authors (hereafter referred to as SB). SB, a 45-year-old male computational neuroscientist, experienced 16 years of progressive symptoms consistent with MAO-A hypofunction producing excess intracellular noradrenaline (NA), serotonin (5HT), and tyramine in the peripheral sympathetic nervous system (Finberg, 2014). Symptoms included constant “fight or flight” response, heart rate and blood pressure spikes after ingesting high-tyramine foods, hyperreflexia, and insomnia, among others. Symptoms were relieved via treatment with either rasagiline or selegiline, which increase MAO-A expression (Inaba-Hasegawa et al., 2013), and adrenergic blockade with carvedilol.

Previously documented MAO-A disorders are universally congenital and have significant developmental and behavioral symptoms, including aggression, such as in Brunner syndrome (Brunner et al., 1993). However, studies have shown that abnormally aggressive behavior in MAO-A disorders is not universal and emerges primarily when a patient has themselves been exposed to childhood violence (Burgess and Peever, 2013).

### Presentation

#### Patient history

Prior to symptom onset, SB had a history of celiac disease, exercise-induced asthma, and ADHD, which were well-controlled with a strict gluten-free diet, extended-release Adderall 20 mg daily (discontinued upon beginning selegiline/rasagiline), and an albuterol inhaler as needed. SB never used use tobacco and ceased moderate alcohol intake after the onset of post-ethanol symptoms and tyramine sensitivity (symptoms 10 and 12, Table 1). His family history included type I diabetes, hypermobile Ehler’s-Danlos syndrome, heart disease, and skin cancer. SB had no criminal history and no history of childhood abuse or significant violent trauma.

**Table 1:** Progression of symptoms with therapeutic interventions.

#	Year and patient age at onset	Symptom complex	Treatment providing effective response and year	Prior pharmacological interventions with partial response
1	2010 Age 31	Insomnia, including difficulty falling asleep and being woken from sleep by symptoms.	Carvedilol Relief first obtained in 2018	Gabapentin, aripiprazole, clonidine
2	2010 Age 31	Constant “fight or flight” response, hypervigilance, increased startle response	Carvedilol Relief first obtained in 2018	Propranolol, aripiprazole, clonidine
3	2010 Age 31	Constant involuntary attentional orienting responses, which neuropsychiatric testing associated with an impact on working memory.	Carvedilol Relief first obtained in 2018	Aripiprazole, clonidine
4	2010 Age 31	Impaired cognition and long-term memory formation.	Carvedilol Relief first obtained in 2018	Aripiprazole, clonidine
5	2010 Age 32	Increased muscle tension, with recurrent spontaneous subluxations and dislocations beginning in 2010 (patella, tempromandibular joint), Spontaneous trismus with dislocation of jaw	Carvedilol and rasagiline or selegiline Relief first obtained in 2020	Clonidine
6	2011 Age 32	Sensation of “restless limb,” urge to move limbs involving extremities, torso, neck.	Carvedilol and rasagiline or selegiline Relief first obtained in 2020	
7	2012 Age 33	Elevated HR and BP (both systolic and diastolic)	Carvedilol, 2018	Clonidine
8	2013 Age 34	Bruxism	Carvedilol and rasagiline or selegiline Relief first obtained in 2020	Clonidine
9	2014 Age 35	Erectile and ejaculatory dysfunction	Carvedilol and rasagiline or selegiline Relief first obtained in 2020	Clonidine
10	2015 Age 36	Euphoria, followed by racing thoughts/anxiety, with onset 8–24 h following rare consumption of ethanol, lasting ~72 h.	Carvedilol and rasagiline or selegiline Relief first obtained in 2020	Abstinence from ethanol
11	2016 Age 38	Dizziness and difficulty with balance	Carvedilol Relief first obtained in 2018	
12	2017 Age 39	HR and BP spikes following consumption of tyramine-rich foods, accompanied by heart palpitations	Carvedilol and rasagiline or selegiline Relief first obtained in 2020	Strict low-tyramine diet
13	2019 Age 41	Difficulty initiating urination	Carvedilol and rasagiline or selegiline Relief first obtained in 2022 under increased dose	

Progression of symptoms with effective treatment and any prior treatments which achieved partial response. Symptoms are presented in order of appearance, noting SB’s age at onset. Successful treatments are presented with the year in which they first provided relief, which does not always follow the order of symptom appearance.

#### Progression of symptoms

SB presented with a constellation of progressive symptoms which accelerated over the course of the illness, characterized by worsening of extant symptoms and emergence of new symptoms (Table 1). Progression accelerated during periods of inadequate sleep and when symptoms were inadequately controlled. When pharmacological control was achieved, progression slowed dramatically. SB reported perceptible short-term reductions in symptom severity following intense cardiovascular exercise, during severe sleep deficits, and when exposed to bright sunlight or a seasonal affective disorder sun lamp. SB’s symptoms were more severe at night, with a circadian cycle of modulation. While many of these symptoms are, on their own, nonspecific, the authors have been unable to identify any extant disorder documented as having presented with this aggregate set of symptoms, particularly the noradrenergic specificity of sympathetic symptoms combined with the distinctive tyramine-induced ‘cheese response’, producing a rise in blood pressure from 116/77 to 149/97 within 30 min of the consumption of aged cheddar cheese (measured in 2020). The tyramine-induced cheese response is specific to inhibited MAO-A activity, and is associated with no other genetic or metabolic disorders (Cassis et al., 1986). Beyond the avoidance of tyramine alleviating the cheese response, dietary changes (including elimination diets, and a steep reduction in foods known to inhibit MAO-A as a consequence of the low-tyramine diet) did not impact the severity of any of the other symptoms listed in Table 1.

#### Exclusion of other diseases

Given the severe impact of these symptoms on SB’s quality of life, a broad work up was pursued. Baseline laboratory studies were within normal limits, including complete blood count, comprehensive metabolic panel, lipid profile, serum aldosterone, renin, cortisol, prolactin, thyroid-stimulating hormone (TSH), free T4, and paraneoplastic antibody panel. Serum adrenaline and taurine were normal, excluding these pathways from the differential.

Pheochromocytoma was excluded via normal fractionated free plasma metanephrine and normetanephrine and abdominal computed tomography (CT) scan. Further imaging studies, including magnetic resonance imaging (MRI) of the brain, cervical, and thoracic spine, were normal. A sleep study could not be completed due to insomnia. Autonomic testing, including heart rate response to deep breathing, blood pressure and heart rate response to Valsalva, sudomotor responses, and tilt table testing, was normal. Whole exome sequencing (WES) was performed in SB and both parents (CentoXome® Trio, CentoMito® Genome Cambridge, USA). No known pathological mutations were identified. This ruled out Fatal Familial Insomnia as well. The sporadic version was ruled out due to persistent lack of patient fatality. An intronic MAOA variant of unknown significance (VUS) was identified (NM_000240.4:c.956134_956133insAACAT; NG_008957.2:g.81405_81406insAACAT) but is unlikely to have direct coding consequence (NP_000231.1:p.?). The same WES ruled out Fatal Familial Insomnia as well. The sporadic version was ruled out due to persistent lack of patient fatality. Psychiatric disorders were excluded after repeated psychiatric evaluations, and symptoms did not improve with reduced life stressors, as with the completion of SB’s graduate studies in 2017, or with trials of standard therapies for anxiety and depression.

Fractionated urine catecholamines showed no elevation in adrenaline, NA, or dopamine (DA) on two separate tests. 24-h urine catecholamines showed normal adrenaline, NA, DA, and vanillylmandelic acid (VMA). NA metabolites (serum normetanephrine, fractionated free metanephrine, and free serum mass) were normal.

Pharmacologically, the following medications were trialed between 2010 and 2018 without significant relief: bupropion, ziprazidone, escitalopram, citalopram, paroxetine, clonazepam, prednisone, fexofenadine, venlafaxine, trazodone, rozerem, tizanidine, and ivermectin.

Clonidine and aripirazole both provided partial relief of several symptoms; however, they were discontinued due to unsustainable side effects. Propranalol and gabapentin provided limited relief and were discontinued when effective management was initiated.

## Materials and methods

### Proposed pathophysiology

MAO has mitochondrially-bound isoenzymes A and B, with distinct physiologic functions (Dingemanse et al., 1998). MAO-A is found in central nervous system and peripheral catecholaminergic neurons, as well as the intestine, vasculature, fibroblasts, and liver. The varied distribution of MAO-A allows for complex modulation of mood, metabolism, and motor functions. MAO-A is involved in intraneuronal catecholamine deamination (Eisenhofer et al., 2004) and has a high affinity for NA, serotonin (5HT), and tyramine (Dingemanse et al., 1998). Previously documented MAO-A deficiencies are all congenital, but there is no mechanistic reason why onset of MAO-A hypofunction could not be triggered later in life. Strong evidence exists for active regulation of MAO-A expression via several pathways, including sirtuin-1 (SIRT-1), the pathway by which rasagiline and selegiline increase expression of MAO-A (Naoi et al., 2015).

**Figure 1:**
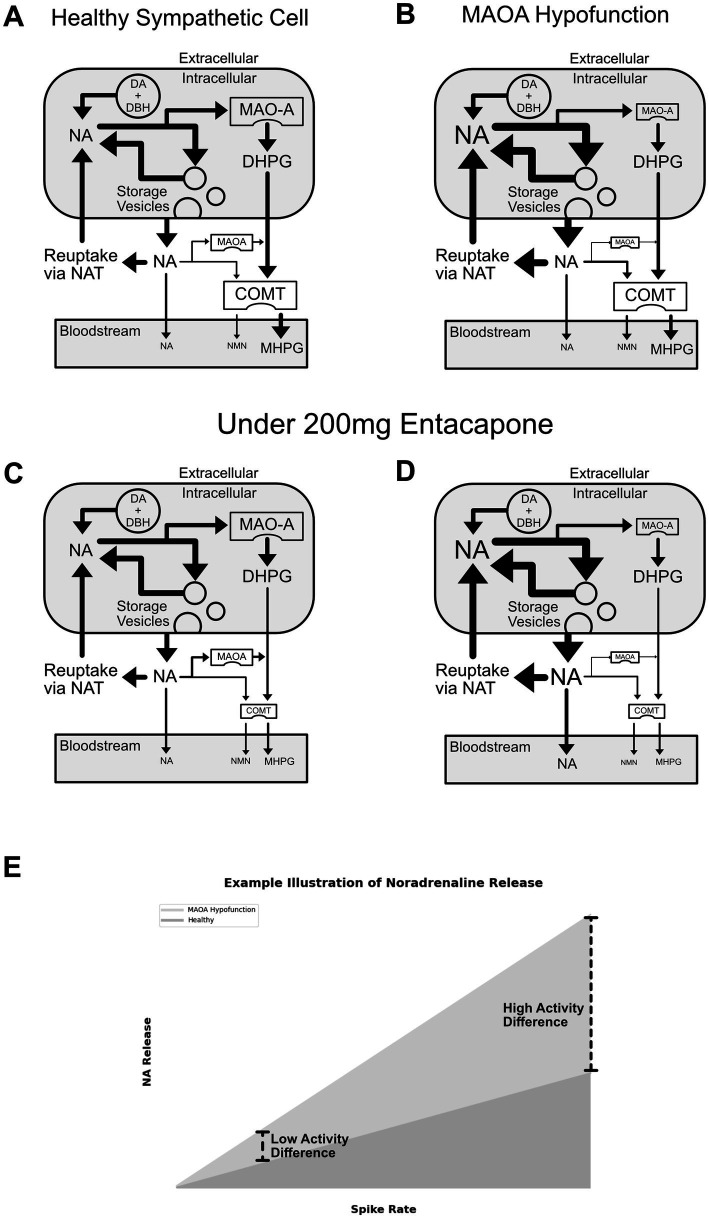
Proposed pathophysiology. Proposed mechanism of pathophysiology, unmedicated and under 200 mg entacapone challenge. **(A–D)** Are relative rate models, featuring quantitative relative alterations in the depicted rates. Baseline rate values are from (Eisenhofer et al., 2001). To preserve visual interpretability, line thickness and font size are scaled proportional to the log_10_ of the assigned numerical values. **(A)** Relative rate model of a sympathetic cell with healthy MAO-A levels. **(B)** Sympathetic cell with MAO-A hypofunction, showing minimal change in plasma noradrenaline (NA), but a notable shift in the ratio of *normetanephrine (*NMN) to *dihydroxyphenylglycol (*DHPG). **(C)** Sympathetic neuron under 200 mg entacapone, no significant change in plasma NA levels. **(D)** Sympathetic neuron with MAOA hypofunction under 200 mg entacapone, showing a significant increase in plasma NA levels. **(E)** Example illustration of synaptically-released NA as a function of spiking activity, showing the multiplicative effects of MAOA hypofunction. DA (dopamine), DBH (dopamine beta-hydroxylase), NA (noradrenaline), DHPG (3,4-dihydroxyphenylglycol), MAO-A (monoamine oxidase A), COMT (catechol-O-methyltransferase), MHPG (3-methoxy-4-hydroxyphenylglycol), MNM (normetanephrine), NAT (norepinephrine transporter).

Progressive MAO-A hypofunction and its downstream effects on NA, serotonin (5HT), and tyramine can elegantly explain all of SB’s symptoms. In healthy human subjects, intracellular NA is largely held in storage vesicles, and the remainder is rapidly degraded by MAO-A. Catecholamine metabolism is also facilitated by catechol-O-methyltransferase (COMT) and sulfotransferase. However, peripheral sympathetic neurons do not express COMT. Therefore, intracellular NA can accumulate in the peripheral nervous system (Finberg and Gillman, 2011). Notably, because of the efficiency of reuptake via NAT, this produces negligible changes in serum NA levels, making it difficult to detect. We do, however, see increase in the NA levels released into the synaptic cleft each time peripheral neuron fires, along with a relative increase in NMN production, alongside a relative decrease in DHPG (Figure 1). This is consistent with the ratio between the two being altered in known conditions of MAO-A dysfunction, as in Brunner Syndrome.

Reduced MAO-A function would be expected to impact 5HT, tyramine, and noradrenaline (NA) metabolism. The resulting increase in sympathetic NA activity produces a unique pattern of downstream effects (Bortolato and Shih, 2011), which remains distinct from general sympathetic overactivity, since only some processes governed by sympathetic innervation are mediated by NA. For example, the sudomotor neurons which innervate sweat glands are almost exclusively cholinergic, and indeed, SB did not exhibit increased sweating. There is evidence of central noradrenergic systems being closely coupled to wakeful postural muscle tone (Ginovart et al., 2006). SB’s increased arousal state described as “fight-or-flight,” insomnia, muscle tension, and bruxism may be associated with an increase in such tone. Urethral contraction (Goldstein et al., 2021), bladder control (Andersson and Arner, 2004), and penile vasoconstriction (Hnoonual et al., 2025) are mediated by NA release from sympathetic neurons, which could explain difficulties initiating urination and erectile dysfunction.

MAO-A hypofunction also affects serotonergic and tyramine pathways. Consumption of ethanol has been shown to affect plasma and urinary levels of MAO-A-derived 5HT metabolites by reducing 5-hydroxyindoleacetic acid (5-HIAA) and increasing 5-hydroxytryptophol (5-HTOL) levels (Helander et al., 1993). Ethanol downregulates the MAO-A 5HT metabolic pathway. In the setting of MAO-A hypofunction, ethanol consumption could plausibly cause resultant symptoms of serotonergic excess including euphoria followed by severe anxiety.

Spikes in heart rate and blood pressure accompanied by heart palpitations following consumption of tyramine-rich foods indicate reduced MAO-A function via the tyramine pathway (Cassis et al., 1986). Tyramine is a naturally occurring amine metabolized by MAO, CYP2D6, and FMO3. When tyramine is ingested in the presence of MAO inhibitors, it displaces NA from vesicular stores. This “cheese reaction” is well-studied in patients on monoamine oxidase inhibitors (MAOIs), which require strict adherence to a low tyramine diet. This diet was an effective treatment for SB’s cardiovascular symptoms prior to initiating rasagiline/selegiline.

There are two primary mechanisms by which MAO-A activity could be reduced, differentiated by their impact on enzyme kinetics. Expression of MAO-A could be decreased, the efficacy of the enzyme could be reduced (e.g., via a reduced binding affinity), or both cold be in play. In the Michaelis-Menton enzyme kinetics model, low expression of MAO-A would reduce V_max_. On the other hand, if only the efficacy MAO-A is reduced, it should increase K_m_. Unfortunately, the precise changes expected *in vivo* under each condition are highly dependent upon other aspects of metabolism and the concentrations of NE and other substances, and therefore we have been unable to determine from extant bloodwork which scenario is occurring in SB.

The reversible MAO-A inhibitor moclobemide partially replicates the effects of a reduction in MAO-A binding affinity, raising K_m_ through competitive inhibition. When administered, moclobemide could be expected to replicate the effects of reduced MAO-A efficacy in healthy adults. In such tests (Illi et al., 1994; Illi et al., 1995), moclobemide reduces levels of DHPG, 3,4-dihydrophenylacetic acid (DHPA), VMA, and homovanillic acid (HVA). Side effects included insomnia, headaches, and dizziness, all of which were among SB’s presenting symptoms.

### Methods: testing of proposed pathophysiology

The following tests of proposed pathophysiology were reviewed by an independent institutional review board (IRB) and were found to not require oversight (PearlIRB #2025–0263). The patient provided informed consent to publication of medical data and waived patient privacy rights. All laboratory tests were conducted by commercial clinical laboratories or in a Clinical Laboratory Improvement Amendments (CLIA) certified laboratory.

#### Methods: entacapone challenge

While initial exclusionary bloodwork showed no elevated NE, EPI or DA plasma catecholamines, this absence was potentially explained by COMT activity, which effects an alternative pathway for noradrenaline breakdown (Eisenhofer et al., 2004). To reduce the confounding impact of COMT, the patient was prescribed entacapone 200 mg, a reversible COMT antagonist (Figure 1). A single dose of entacapone 200 mg inhibits COMT enzymatic activity by 65%, reaching maximum effect within 30 min of oral administration (Männistö et al., 2024; Jian et al., 2021; Keränen et al., 1993). However, healthy human subjects do not display a change in plasma NE even when very high doses of entacapone are administered due to compensatory MAO-A activity. (Koulu et al., 1989). After a tapered cessation of rasagiline and administration of entacapone, a baseline blood sample was acquired and then 200 mg of entacapone was orally administered. SB then remained seated and refrained from significant mental or physical activity for the next 2 hours, whereupon a second blood sample was taken. The blood samples were tested for plasma NE levels as well as free normetanephrines (NMN). This procedure was then repeated six times following resumption of rasagiline.

#### Methods: Deaminated catechol measurement

The most direct test performed was measurement of deaminated catechols (catecholamine metabolites) performed non-experimentally at a CLIA certified laboratory at the National Institutes of Health. After tapering off all prior medications until symptoms re-emerged and stabilized, controlling blood pressure with amlodipine, a resting antecubital blood sample was obtained and plasma was separated by centrifuge at 4 °C, 4000 rpm for 15 min (Lachoi Centrifuge). Samples were stored at −80 °C using a portable freezer (Stirling Model Ult25NEU Portable freezer) and then packed in dry ice and shipped immediately to the laboratory. There, the samples were thawed and underwent alumina extraction of catechols (Holmes et al., 1994) and were processed using a pump and refrigerated autosampler connected to a reversed-phase liquid chromatography column, kept at constant temperature using a column jacket. The column effluent was then passed through an electrochemical detection system, and measurements of dihydroxyphenylglycol (DHPG), NE, dihydroxyphenylalanine (DOPA), epinephrine (EPI), DA, and dihydroxyphenalyacetic acid (DOPAC) were obtained.

#### Methods: comparison with X-linked MAO-A microdeletion

To contextualize SB’s biochemical abnormalities, we compared his quantitative blood test values with previously published studies of patients with X-linked MAO-A microdeletion (Lenders et al., 1996). Comparisons were made with two Brunner syndrome patients and with a population of healthy control subjects, examining values for dihydroxyphenylglycol (DHPG), noradrenaline (NA), dihydroxyphenylalanine (DOPA), adrenaline (ADR), dopamine (DA), and dihydroxyphenlyacetic acid (DOPAC), free or non-sulfate conjugated normetanephrine (NMN) and the calculated ratio of NMN: DHPG.

### Methods: pharmacological management and response

Cognizant of concerns that a patient acting as co-author might unduly bias his own treatment, we wish to clarify that all treatment in this section was initially prescribed prior to establishing care with co-author Erik Armitano, MD in 2022. Effective treatment had at that point been established under physician-patient relationships with other physicians with whom SB had no professional association. This ensured that any treatments SB proposed were backed by sufficient evidence to pass unbiased scrutiny.

Effective treatment informed by the proposed pathophysiology consisted of the combined off-label use of two currently FDA-approved pharmacological agents: either rasagiline or selegiline, which increase MAO-A expression (Inaba-Hasegawa et al., 2013), and carvedilol for alpha-1/non-cardioselective beta-adrenergic blockade.

#### Methods: carvedilol—noradrenergic blockade

Carvedilol is a nonselective alpha1- and beta-adrenergic blocker, which prevents binding of NA to its receptor. Following clonidine discontinuation, the patient was prescribed 6.25 mg carvedilol twice per day in 2018. This was converted to 20 mg extended release in the same year and has since been adjusted as needed to control symptoms.

#### Methods: rasagiline/selegiline—MAO-A modulation

Rasagiline and selegiline are selective MAO-B inhibitors which have been shown to upregulate MAO-A expression in human subjects via Sp1/KLE11 signaling [reviewed in (Naoi et al., 2015)]. In 2020, dextroamphetamine prescribed for the patient’s attention deficit disorder was discontinued and rasagiline (MAO-B inhibitor/MAO-A up-regulator) was prescribed and transitioned to selegiline in 2024 at the request of insurance.

#### Ruling out habituation

To rule out habituation as an explanation for required dosage increases, in 2020—prior to the initiation of rasagiline—SB executed a month-long taper and cessation of treatment with carvedilol. Blood pressure was controlled using amlodipine (5 mg/day), a calcium channel blocking anti-hypertensive, after SB reached stage II hypertension. Once the taper was complete, he underwent formal autonomic reflex testing at Brigham & Women’s Hospital in Boston, USA and subsequently titrated back to a dose of carvedilol sufficient to achieve symptom control.

## Results

### Results: tests of proposed pathophysiology

#### Results: entacapone challenge

After a tapered cessation of rasagiline and administration of entacapone, SB’s plasma NA increased by 89%. When repeated on concomitant rasagiline, NA increased by a mean of 19.7% (standard deviation 37.50%). Notably, the percentage change under entacapone is reduced when the dose of rasagiline is increased (see Table 2).

**Table 2:** Noradrenaline levels (pg/mL) before and after entacapone 200 mg oral challenge.

Challenge and date	Challenge No.1 12–2021	Challenge No.2 04–2022	Challenge No.3 05–2022	Challenge No.4 12–2022	Challenge No.5 01–2023	Challenge No.6 02–2023	Challenge No.7 06–2023
NA pre-entacapone	244	400	559	236	430	494 L	753
NA post-entacapone	807	456	718*	447	433	402	786
Concomitant rasagiline	No	Yes (9 mg)	Yes (9 mg)	Yes (9 mg)	Yes (10 mg)	Yes (10 mg)	Yes (10 mg)
Notes			Post-entacapone medical record not available				

Entacapone challenges, with pre- and post- entacapone serum NA levels, the amount of rasagiline administration at the time, and any additional notes relevant to each challenge.

#### Results: deaminated catechol measurement

DHPG and DOPAC, catecholamine by-products of MAO-A metabolism, were found to be significantly reduced (286 pg./mL (normal value 500–1,400 pg./mL) and 957 pg./mL, (normal value 1,000–3,000 pg./mL) respectively (Table 3). This confirmed an abnormally low level of MAO-A activity. (Mejia et al., 2001)

**Table 3:** Comparative plasma concentrations (pg/mL) of catecholamines and metabolites in MAO-deficient subjects and in control subjects.

Case subject	Deficiency	DHPG	NA	DOPA	ADR	DA	DOPAC	Free NMN	Free NMN: DHPG ratio
Normal range	None	500–1,400	80–498	900–2,500	4–83	<46	1,000–3,000		
SB	MAO-A hypofunction	**286**	272	1,169	47	15	**957**	**221 (6 months prior)**	**Estimated at 0.78**
Patient No.3 [from Lenders et al. (1996)]	X-linked MAO-A	**93**	151	N/A	18	3	**672**	**218.01**	**2.34**
Patient No.4 [from Lenders et al. (1996)]	X-linked MAO-A	**70**	354	N/A	7	5	**622**	**415.86**	**5.94**
Control subjects [from Lenders et al. (1996)]	None	1,014.1 ± 501.5	228 ± 199.6	N/A	0	0	1,530.2 ± 1,883.3	45.8 ± 40.3	0.0036 to 0.17

Table of measured (for our patient) and reported [for patients and controls from Lenders et al. (Lenders et al., 1996)] values for dihydroxyphenylglycol (DHPG), noradrenaline (NA), dihydroxyphenylalanine (DOPA), adrenaline (ADR), dopamine (DA), and dihydroxyphenlyacetic acid (DOPAC), free or non-sulfate conjugated normetanephrine (NMN) and the calculated ratio of NMN: DHPG. All values are converted from original units to pg./mL for comparison. Lenders originally reported their grouped control subject values as a mean and standard deviation. Here, we provide a range of DHPG and NMN values, indicated by the mean as well as upper and lower expected bounds (mean ± two times the standard deviation). The ratio of NMN: DHPG for control the grouped control subjects is also reported as an expected range, with the lowest expected ratio being calculated from the lower expected bound of NMN and the upper expected bound of DHPG, and the highest expected ratio doing the opposite. Note that for our patient, NMN measurements were not taken at the same time as DHPG, so we used the most recent available NMN measurements to produce an estimated ratio.

#### Results: comparison with X-linked MAO-A microdeletion

In comparison with published data from X-linked MAO-A microdeletion (Lenders et al., 1996), we found similar biochemical abnormalities in our patient (Table 3). Both our patient and the X-linked MAO-A group had reduced plasma DPHG and DOPAC. While our patient’s NMN was not measured at the same time as the DHPG measurement, the baseline plasma value measured during entacapone challenge #7 (performed 6 months prior) was used to estimate the ratio of free NMN to DHPG. This estimated free NMN to DHPG ratio in our patient was 0.78 while the two MAO-A X-linked patients also had similar ratios of 2.33 and 5.9 (patient 3 and 4) respectively. In contrast, the free NMN: DHPG ratio in the control group was 0.045 and even when maximum ranges were used the maximum ratio was 0.17. Generally, SB displays the same deviations from controls as Brunner syndrome patients, but at lower amplitudes.

### Results: pharmacological management and response

On combination therapy with carvedilol and rasagiline/selegiline, SB has achieved complete relief of all symptoms delineated in Table 1. This clinical response to treatment with carvedilol and either rasagiline or selegiline is consistent with progressive MAO-A hypofunction.

#### Results: carvedilol—noradrenergic blockade

Carvedilol provided significant relief of a subset of symptoms (Table 1) - and the patient reported no side effects. Relief was of short duration on the immediate release version, with symptoms returning 3 hours after administration. Extended-release doses twice per day were able to provide continuous relief. After 6 months the controlled symptoms began to re-emerge, and dosage was titrated until control of the re-emerging symptoms was achieved. This pattern has repeated in a stepwise fashion, with symptom severity progressing rapidly when uncontrolled, and quite slowly once a therapeutic dosing is achieved. Despite the maximum dosing recommendations of carvedilol, the patient consistently responded to supramaximal dosages without any known side-effects, including orthostatic intolerance, bradycardia, fatigue, or syncope. The amount of carvedilol needed to manage symptoms has consistently maintained the patient’s blood pressure in mid-to-upper normal ranges.

#### Results: rasagiline/selegiline—MAO-A modulation

Rasagiline (and later selegiline) was found to be effective at supramaximum dosing with further additional control of symptoms (Table 1) and without adverse side effects. Interestingly, despite ADHD symptom severity increasing during the cessation of dextroamphetamine, the patient noted significant relief from ADHD symptoms and reported symptoms returning coincident with the requirement for increased dosing of rasagiline.

Required carvedilol dosing was reduced by 40% from 200 mg daily to 120 mg daily, suggesting that upregulation of MAO-A reduced the need for adrenergic blockade. Additionally, the plateau period of the stepwise symptom progression noted above was significantly extended by joint administration of carvedilol and rasagiline/selegiline.

As of writing, SB is on carvedilol ER 160 mg twice per day (320 mg/day), and selegiline HCL 15 mg twice daily (30 mg/day), having been transitioned to an equivalent dose from rasagiline at the request of insurance. Blood pressure and heart rate remain normal, with no indications of hypotension, and the patient reports no side effects. SB’s symptoms are well controlled.

#### Results: ruling out habituation

Symptoms returned during the taper and cessation. Autonomic testing was normal. However, upon titration back onto carvedilol the dosing requirement was higher than previously needed to control the symptoms, suggesting that the patient had not habituated to this medication.

## Discussion

### Summary

Here we present a patient with a constellation of symptoms consistent with a previously undocumented possible novel adult-onset, progressive MAO-A hypofunction. We have provided a plausible mechanistic pathway backed by physiologic and pharmacologic evidence for progressive MAO-A hypofunction as the underlying cause of SB’s clinical syndrome.

### Potential bias and mitigation

We are aware that SB’s role as both patient and author is a rarity in medical literature, but it is not unprecedented (JNK et al., 2023) We have taken pains to anticipate and mitigate potential bias, as well as relying as heavily as possible on quantitative, empirical tests to confirm the hypothesis of MAO-A hypofunction. There can be bias in test selection, but we strove to ensure that all standard tests were performed, and the reliance on independent physicians for initiation of treatment ensured that sufficient evidence had been gathered and other possibilities ruled out prior to any trial of novel alternative treatments. We also recognize the inherent ethical concerns of having a patient as co-author. In order to comply with accepted ethical practices, all approaches reported herein were examined by an independent institutional review board (PearlIRB), which flagged no ethical concerns in its findings.

### Comparison with previously documented disorders

Our reported laboratory findings trend in the direction of values found in previously published studies (Lenders et al., 1996) on X-linked MAO-A hypofunction and highlight the utility of plasma NM: DHPG ratio in determining MAO-A enzyme dysfunction in adults even when the disorder is less severe than in Brunner syndrome. The clinical phenotype observed in SB also trends in the same direction with reduced severity, except in areas which may be explained by the delayed onset.

The authors hypothesize that many behavioral aspects were avoided in our patient by the delay of clinical onset until after neurodevelopmental stages were completed. SB displayed no developmental deficits, and has no history of violent crime, despite reporting struggles with impulsive fight or flight reactions during periods of absent or ineffective medication. The lack of violence in SB’s upbringing could explain the absence of criminal aggression (Burgess and Peever, 2013).

Previously described MAO-A disorders have also not been progressive. In contrast, SB’s symptoms grew worse over time in a stepwise fashion plateauing once a therapeutic dosing is achieved. This suggests that some aspect of the expression of symptoms interacted with the disease in a feedback loop, driving an increase in severity.

### Future directions

#### Possible affected population

Further investigation is warranted on several fronts. The prevalence of this disorder is unknown but given the difficult path to confirmation and successful treatment experienced by SB, we caution against any assumption that a lack of previous reporting indicates rarity. A population suffering from this disorder could plausibly make up a sizeable percentage of autonomic patients who suffer sympathetically mediated symptoms but do not meet the diagnostic criteria for PTSD, generalized anxiety disorder (GAD), or postural orthostatic tachycardia syndrome (POTS). With confirmation available via blood tests (see below), other patients displaying a similar pattern of symptoms could be tested, and an estimate of the affected population size might be obtained.

#### Further diagnostic testing

Measurements of plasma catechol’s in a CLIA-certified laboratory played a critical role in diagnosing MAO-A (and potentially MAO-B or MAO-AB) dysfunction without requiring SB’s enrollment in an experimental protocol. Such measurements, being available in only a small number of labs, are not widely commercially accessible to patients. We demonstrate the feasibility of outpatient sample collection, but without widespread commercial availability the identification of additional patients is likely to rely on enrollment of prospective patients in experimental protocols. In addition to DHPG measurement, several complementary experimental methodologies exist which and could facilitate diagnosis and potentially offer further understanding of the underlying mechanism. MAO assays using Fibroblast cultures were used in the initial discovery of Brunner Syndrome (Brunner et al., 1993), and have been applied more recently in the functional confirmation of MAO-A variants (Oliver et al., 2022). Positron Emission Tomography with [11C]-Harmine (a competitive and reversible inhibitor of MAO-A) allows for in-vivo measurements of MAO-A activity in the human brain (TMA et al., 2021). Fluorescence PCR (Simonsen et al., 2002) has shown efficacy in genotyping alleles of interest. Westernblot techniques (Thor, 2003) can measure expression in tissue samples, or potentially even macrophages, and finally small molecule fluorescent probes (Jian et al., 2021) can allow measurement of monoamine oxidase-A and monoamine oxidase-B expression *in vivo*.

#### Further investigation into mechanism

While the underlying mechanism has been confirmed as MAO-A hypofunction, it remains unknown whether the hypofunction arises from a low-functioning variant in the MAO-A enzyme or from under expression of the MAO-A gene. Either one could account for the results, and further investigation via laboratory testing could clarify the situation considerably or even suggest more effective methods of treatment. While current tests have not offered the ability to differentiate the two possible cases, tests such as those in (Oliver et al., 2022) allow more direct measurement of enzymatic kinetic parameters.

Although SB has a documented VUS in the MAOA coding region, it is possible that his symptoms are not due to a genetic mutation. Epigenetic changes such as DNA methylation, histone modification, and chromatin remodeling are heritable chemical changes that alter gene expression without changing the underlying genetic sequence. Epigenetic changes could potentially account for low-functioning of a normal MAOA gene, or even a progressively increasing sensitivity to environmental inhibitors of MAO-A. Alternatively, under expression could be assessed by measuring messenger ribonucleic acid (mRNA) levels in peripheral neurons.

## Conclusions and implications for practitioners

This report presents compelling evidence that the patient’s symptoms are consistent with a previously undocumented novel adult-onset, progressive MAO-A hypofunction. More broadly, it demonstrates the potential for patients to suffer from illnesses which cannot readily be detected by commonly available tests and highlights the use of advanced biochemical analysis to aid in diagnosis and treatment. The authors recommend that physicians consider these conclusions when assessing hyperadrenergic syndromes whose etiology eludes categorization.

## Data Availability

The datasets presented in this article are not readily available because of ethical and privacy restrictions. Requests to access the datasets should be directed to the corresponding author.

## Author’s note

While S. Berteau was affiliated with several institutions during the time this work occurred, it was performed independently.
