# Correction: Case Report: A possible novel adult-onset, progressive MAO-A hypofunction

**DOI:** 10.3389/fnins.2026.1901238

**Published:** 2026-06-17

**Authors:** Stefan Berteau, Erik Armitano

**Affiliations:** 1 Independent Researcher, Edinburgh, United Kingdom; 2 Complex Autonomic Center, Mountlake Terrace, WA, United States

**Keywords:** carvedilol, hypofunction, MAO-A, monoamine oxidase A, norepinephrine, plasma catechols, rasagiline

The reference citation for Lenders et al., 1996 was erroneously written as Männistö et al., 2024. It should be Lenders et al., 1996, and the correct full reference is:

Lenders, J. W., Eisenhofer, G., Abeling, N. G., Berger, W., Murphy, D. L., Konings, C. H., et al. (1996). Specific genetic deficiencies of the A and B isoenzymes of monoamine oxidase are characterized by distinct neurochemical and clinical phenotypes. *J. Clin. Invest*. 97, 1010–1019. doi: 10.1172/JCI118492

The reference citation for Männistö et al., 2024 was erroneously written as Inaba-Hasegawa et al., 2013. It should be Männistö et al., 2024.

The reference citation for Inaba-Hasegawa et al., 2013 was erroneously written as Naoi et al., 2015. It should be Inaba-Hasegawa et al., 2013.

The reference citation for Naoi et al., 2015 was erroneously written as Eisenhofer et al., 2004. It should be Naoi et al., 2015, and the correct full reference is:

Naoi, M., Riederer, P., and Maruyama, W. (2015). Modulation of monoamine oxidase (MAO) expression in neuropsychiatric disorders: genetic and environmental factors involved in type A MAO expression. *J. Neural Transm*. 123, 91–106. doi: 10.1007/s00702-014-1362-4

The reference citation for Eisenhofer et al., 2004 was erroneously written as Eisenhofer et al., 2001. It should be Eisenhofer et al., 2004.

The reference citation for Eisenhofer et al., 2001 was erroneously written as Finberg, 2014. It should be Eisenhofer et al. 2001.

The reference citation for Finberg, 2014 was erroneously written as Andersson and Arner, 2004. It should be Finberg, 2014.

The reference citation for Andersson and Arner, 2004 was erroneously written as Helander et al., 1993. It should be Andersson and Arner, 2004.

The reference citation for Helander et al., 1993 was erroneously written as Holmes et al., 1994. It should be Helander et al., 1993, and the correct full reference is:

Helander, A., Beck, O., Jacobsson, G., Löwenmo, C., and Wikström, T. (1993). Time course of ethanol-induced changes in serotonin metabolism. *Life Sci*. 53, 847–855. doi: 10.1016/0024-3205(93)90507-y

The reference citation for Holmes et al., 1994 was erroneously written as Lenders et al., 1996a. It should be Holmes et al., 1994.

The original version of this article has been updated.

